# Crossmodal Discrimination of 2 vs. 4 Objects across Touch and Vision in 5-Month-Old Infants

**DOI:** 10.1371/journal.pone.0120868

**Published:** 2015-03-23

**Authors:** Aurélie Coubart, Arlette Streri, Maria Dolores de Hevia, Véronique Izard

**Affiliations:** 1 Université Paris Descartes, Sorbonne Paris Cité, Paris, France; 2 CNRS UMR 8242, Laboratoire Psychologie de la Perception, Paris, France; University of Padova, ITALY

## Abstract

Infants are known to possess two different cognitive systems to encode numerical information. The first system encodes approximate numerosities, has no known upper limit and is functional from birth on. The second system relies on infants’ ability to track up to 3 objects in parallel, and enables them to represent exact numerosity for such small sets. It is unclear, however, whether infants may be able to represent numerosities from all ranges in a common format. In various studies, infants failed to discriminate a small vs. a large numerosity (e.g., 2 vs. 4, 3 vs. 6), although more recent studies presented evidence that infants can succeed at these discriminations in some situations. Here, we used a transfer paradigm between the tactile and visual modalities in 5-month-olds, assuming that such cross-modal paradigm may promote access to abstract representations of numerosities, continuous across the small and large ranges. Infants were first familiarized with 2 to 4 objects in the tactile modality, and subsequently tested for their preference between 2 vs. 4, or 3 vs. 6 visual objects. Results were mixed, with only partial evidence that infants may have transferred numerical information across modalities. Implications on 5-month-old infants’ ability to represent small and large numerosities in a single or in separate formats are discussed.

## Introduction

In the last few decades, a wealth of studies have investigated the representation of numerosity in infants. These studies showed that preverbal infants possess two cognitive systems capable of encoding numerical information [1–2]. The first system, the Approximate Number System (ANS), encodes numerosities in an approximate way. The second system relies on parallel individuation to track objects in small sets, and by doing so, also encodes the set numerosity.

The ANS has been proved to exist from birth on [3–4] and is present throughout life. ANS representations of numerosity are approximate, so that infants will only discriminate two numerosities if they are sufficiently different in terms of ratio. Infants’ acuity with numerosity gets finer with age: newborns need a 3:1 ratio to discriminate large numerosities (for example: they discriminate 4 vs. 12, 6 vs. 18, but not 4 vs. 8 [3]), but by 4.5 to 6 months of age, infants can discriminate numerosities in a 2:1 ratio [5–6].

Contrary to large numerosities, the system tracking small sets is not constrained by ratio, and instead presents two characteristic signatures. First, even young infants can make precise discriminations such as 2 vs. 3 items, at an age where a 2:3 ratio does not lead to discrimination of large numerosities. For example, Féron, Gentaz & Streri [7] showed that 5-month-old infants successfully discriminate 2 vs. 3 objects using a transfer paradigm from the tactile to the visual modality. In this study, after being familiarized with either 2 or 3 objects in the tactile modality, infants looked longer at a visual display containing a new number of objects (respectively 3 or 2). The second signature of this system is a constraint based on set size: infants can only track sets containing up to 3 objects, and beyond this limit, they fail to even encode that there is more than 1 object in the set (set size signature; [8]). For example, after having seen 1, 2 or 3 objects hidden in a box, 10- to 12-month-old infants continue to search in the box until they find the exact number of objects. However, if 4 objects were initially hidden in the box, infants stop searching after having retrieved only 1 object. This same set size signature was also observed in a second task, where infants made choices between two buckets that contained different numbers of crackers [9].

More generally, infants often fail to discriminate small vs. large numerosities, even when the ratio is favorable [4–8–10–11]. This failure was mainly observed in the visual modality. Xu [11] showed that in conditions where infants succeeded at discriminating between two large numerosities (4 vs. 8), they failed when presented with a contrast of a small vs. a large numerosity (2 vs. 4); a failure that was later extended to another pair of numerosities in a 2:1 ratio (3 vs. 6 [10]). Several interpretations of this result are possible. First, the ANS may not be able to encode small numerosities, such that only the system for small sets would be available to represent numerosity for sets of 1–3 objects, thus making comparisons between small and large numerosities impossible. Second, the ANS may encode all numerosities with no discontinuity between small and large ranges; however, the representations elicited by the small sets system would be more salient and thus would be masking ANS representations for small numerosities.

Suggestively, in adults and animals ANS representations are available for large as well as small numerosities. For example, Burr, Turi & Anobile [12] showed that when attentional resources are engaged by a second, non-numerical task, adults revert to ANS representations in the small range of numerosities, with a characteristic ratio signature. Similarly, in animals, Jones & Brannon [13] adapted Feigenson’s infant cracker task to adult primates, and did not observe any discontinuity between the small and large ranges. Primates were able to compare a small vs. a large set; moreover, for all ranges of numerosities, their behavior was constrained by ratio, thus showing that they encoded all numerosities in the format of analogue magnitudes. This latter result is further supported by data from electrophysiological studies in monkeys, which show no discontinuity across the small and large numerical ranges [14–15].

In line with the hypothesis of continuity, recent results suggest that infants are able to compare a small vs. a large numerosity in some situations. For example Cordes & Brannon [10] showed that in conditions where infants fail at discriminating a small vs. a large numerosity, they succeed with a ratio that is twice the minimum within-range ratio for this age (ratio of 1:4 instead of 1:2). Success at small vs. large numerosity discriminations was also observed in conditions where the load on attentional resources was high [16]. Using a change detection paradigm, Starr et al. [16] observed that infants looked preferentially at a stream of rapidly presented visual arrays changing in numerosity, over another stream where numerosity was constant. This preference was independent of the size of the sets presented, but was constrained by the ratio of the numerosity change: six-month olds detected the changing stream only if the numerosity changed in a 2:1 ratio, even when the numerosities crossed the small-large divide (2 vs. 4), while they failed with a 2:3 ratio, even when presented with small sets (2 vs. 3). Lastly, whereas failures to discriminate small vs. large visual arrays have been reported in several studies, it is not clear whether this pattern extends to other modalities. Mixed results have been observed for example in the auditory modality, one study reporting a discontinuity between the small and large ranges (in particular: success at 2 vs. 3, failure at 2 vs. 4 [17]), and another reporting ratio-based discrimination across the whole range of numerosities tested (in particular: success at 2 vs. 4, failure at 2 vs. 3 [18]). These discrepancies may be caused by an ambiguity inherent to auditory stimuli. While two visual items necessarily signal two objects, two tones can have been emitted by a single object or agent, and this ambiguity may have modulated the engagement of the object tracking system across studies.

Collectively, these three lines of results suggest that in infants, just as in animals and adults, the ANS may be functional across the whole range of large and small numerosities. Whereas small sets’ representations are more salient and drive behavior in some experimental situations, presenting numerosities separated by a large ratio, presenting stimuli at a fast pace, or using auditory/sequential stimuli could disengage infants from object tracking representations, giving them access to ANS representations of small numerosities. Here, we tested whether bimodal situations could also incite infants to represent small numerosities in the format of ANS, and therefore lead to successful discriminations of a small vs. a large numerosity. Indeed, bimodal situations might promote access to more abstract representations [19], just as increasing variability in a habituation phase can enable infants to encode more abstract features of stimuli [20]. We thus reasoned that infants experiencing sets across two modalities may be more prone to represent the sets numerosities in term of ANS representations, since ANS representations present a higher level of abstraction than object tracking representations—the ANS, unlike object tracking, abstracts away objects locations and features.

We used a tactile to visual transfer paradigm developed by Féron et al [7], adapting it to test a contrast between a small (2) and a large (4) numerosity. Five-month-old infants were familiarized in the tactile modality with either 2 or 4 objects and then tested in the visual modality with 2 and 4 objects. Our predictions are threefold. First, if 5-month-old infants are able to access a single format of representations for both small and large numerosities in this transfer paradigm, we should obtain a general preference for the novel numerosity, as was found in Féron et al.’s original study. Second, if infants encode the small and large sets using different formats, they should fail to transfer numerosity across the tactile and visual phases of the experiment, and no preference should be observed. A third possibility can be envisaged. While the visual stimuli may be compatible with either formats of representation, it is possible that the slow-paced, sequential presentation used in the tactile familiarization phase can only be processed through object tracking representations. In this case, infants familiarized to 2 items may still display a preference for the novel numerosity (4) at test, while infants familiarized to 4 items would not be able to encode any numerical information, and therefore would not display a preference for the novel numerosity (2).

## Experiment 1: Cross-Modal Transfer of 2 vs. 4 Objects across the Visual and Tactile Modalities

### Method

#### Participants

32 infants (16 females) from 4 months and 16 days to 5 months and 4 days participated in this experiment (mean age: 4 months and 23 days). The data of 12 infants were excluded because of: crying (2), experimental error (2), intervention of the parents (2) or poor camera angle (6).

#### Ethics statement

The experiment was conducted after obtaining approval from the “Comité de Protection des Personnes—Ile-de-France 2” (IRB 00001072). A written informed consent was obtained from a legal guardian of each infant.

#### Stimuli

**Fig 1:**
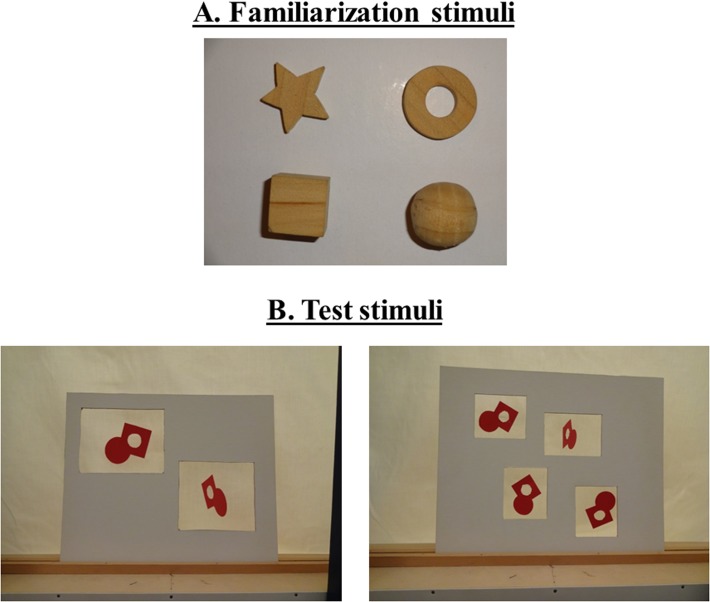
Stimuli. A. Stimuli used in the tactile familiarization. When familiarized with 2 objects, only the cube and the ring were used. B. Visual arrays presented in the test phase.

The stimuli were created following the methods of Féron et al. [7]. The objects used for the tactile familiarization were 4 wooden objects: a ring (diameter 2.4 cm), a sphere (diameter 2 cm), a cube (side 1.7 cm), and a star (diameter 2.5 cm) (Fig. 1.A). In the condition of familiarization to 2 objects, only the cube and the ring were used.

The visual arrays presented in the test phase consisted in boards with either 2 or 4 holes in which the objects were hung (Fig. 1.B). The visual objects retained some features of the objects presented in the tactile familiarization (curves, angles, hole), but nonetheless differed from the familiarization objects, so that the task could not be solved by recognizing objects shapes. The arrays used in the visual test phase contained either 2 or 4 such identical objects. In order to control for cumulative area, the objects presented in the 2-item array were 2 times larger than the objects presented in the 4-item array, so that the total surface of the 2 objects was 47.84 cm^2^ (23.92 cm^2^ per object), and that of the 4 objects was 48.4 cm^2^ (12.1 cm^2^ per object).

#### Experimental set-up

Infants were placed in a baby seat facing a stage made of white boards. During the tactile familiarization phase, a large white bib was attached to the neck of the baby on one side and above the stage on the other side, so that the infant could not see his/her hand holding the objects. The bib was removed during the test phase, allowing the infant to see the stage with the visual displays. The stage was equipped with a curtain that could open and close to mark the beginning and the end of each trial. A video camera placed under the visual display recorded the infant’s face.

#### Procedure

The experiment started with a tactile familiarization phase, during which either 2 or 4 objects were sequentially placed in the right hand of the infant. The right hand was used because infants’ grasping is stronger and lasts longer with the dominant hand at this age [21]. To equalize the total duration of the tactile familiarization across the two conditions (2 or 4 objects), the experimenter ensured that infants hold each object during 45 seconds when familiarized to 2 objects, and during 22 seconds when familiarized to 4 objects. After the tactile familiarization, infants received 6 test trials, showing visual arrays with either 2 or 4 objects in alternation. Test trials began when the experimenter opened the curtain and ended when the infant stopped looking at the display for 1 second, after at least 1 second of looking time had accumulated.

#### Data recording and analysis

Looking times were assessed offline by another observer, blind to experimental conditions. For 62.5% of the infants, online looking times were saved, and reliability between online and offline coding was high (r = 0.99). The analyses presented below are based on the offline coding only.

Data were analyzed in an ANOVA with the two between-subject factors of Condition of familiarization (2 or 4 tactile objects) and Order of visual test presentation (2 visual objects presented first or 4 visual objects presented first), and the two within-subject factors of Test type (2 objects or 4 objects) and Test pair (first, second or third).

### Results

**Fig 2:**
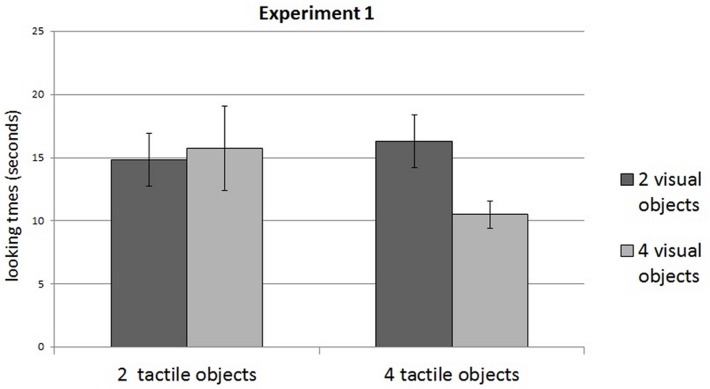
Results of Experiment 1. Infants looked longer at 2 visual objects after being familiarized with 4 tactile objects, but presented no visual preference when familiarized with 2 tactile objects. Error bars are s.e.m.

The analyses revealed a significant interaction between Condition of familiarization and Test Type (F(1,28) = 7.06, p = .012; familiarization with 2 objects, test 2 objects m = 14.84 s, test 4 objects m = 15.75 s; familiarization with 4 objects, test 2 objects m = 16.29 s, test 4 objects m = 10.50 s). Post-hoc analyses revealed that infants familiarized with 4 objects looked longer at visual arrays of 2 objects (13 infants looked longer at the 2-object array, 1 infant looked longer at the 4-object array, and 2 infants looked equally long at both displays (less than 1 s difference); p = .003), while infants familiarized with 2 objects looked equally long at both displays (8 infants looked longer at the 2-object array, 6 looked longer at the 4-object array, and 2 infants looked equally long at both displays; p = .61) (Fig. 2).

Besides the main interaction, we also observed an effect of Test pair (F(2,56) = 8,87, p = .0004) together with an interaction between Test pair and Condition of familiarization (F(2,56) = 3.97, p = .02): infants looked generally longer at the first Test Pair (the two first visual arrays), and especially when they had been familiarized with 2 objects.

### Discussion

A previous study using the same tactile-visual transfer paradigm with small sets (2 vs. 3 items) found 5-month-old infants to look longer to visual displays that were novel in numerosity, after being familiarized to hold 2 or 3 objects [7]. Here, we tested a contrast between a small (2) and a large (4) numerosity with the same methods, and found the same interaction. At first view, this result appears in line with recent findings, where infants were able to compare a small vs. a large numerosity: perhaps, our cross-modal paradigm triggered infants to represent all numerosities in a generic format, applicable to both ranges.

In detail, however, the preference for the novel numerosity was found only in the group of infants familiarized with 4 objects, while the infants familiarized with 2 objects displayed no preference. This pattern was unexpected. Given that sequential presentations usually engage the small sets system, at least in the visual modality [2], and given Féron et al.’s positive results for a tactile familiarization to 2 objects, we would have predicted that infants familiarized to a small numerosity (2) had more chances to succeed than the infants familiarized to a large numerosity (4). It is however possible that the preference for 2-item arrays displayed after familiarization with 4 objects did not reflect a true reaction to novelty. Instead, perhaps infants have a spontaneous preference for our 2-objets visual array over the 4-object array, such that what appeared to be a novelty preference after familiarization with 4 objects may be just an expression of this spontaneous preference. At the same time, a spontaneous preference for 2-item arrays would have masked infants’ reaction to novelty in the group familiarized to 2 objects. To test for this possibility, we ran a control baseline experiment in which we measured infants’ spontaneous preference for arrays containing 2 vs. 4 objects, with no previous familiarization phase.

## Experiment 2: Spontaneous Preference for 2 vs 4 Visual Objects

In order to further investigate the pattern of results observed in Experiment 1, the same visual arrays of 2 and 4 objects were presented to a new group of 5-month-old infants, without previous tactile familiarization.

### Method

#### Participants

16 infants (10 females) from 4 months and 17 days to 5 months and 0 days participated in this experiment (mean age: 4 months and 23 days). The data of 2 infants were excluded because of video equipment failure (1), and intervention of the experimenter (1).

#### Procedure

The same visual displays of 2 and 4 objects were presented in 6 trials in alternation, just as in Experiment 1. The set-up was identical to Experiment 1, except that we did not attach the bib around the neck of the infant, since this experiment did not include a tactile familiarization phase.

#### Data recording and analysis

Looking times for analyses were measured offline from the video recording. Reliability between the online and the offline coders was high (100% of the infants, r = 0.95).

Data were analyzed in an ANOVA with the one between-subject factor of Order of visual test presentation (2 visual objects presented first or 4 visual objects presented first), and the two within-subject factors of Test numerosity (2 objects or 4 objects) and Test pair (first, second or third). In addition to the main ANOVA, we ran two other ANOVAs in order to compare the Baseline experiment to respectively the Familiarization to 2 objects and to the Familiarization to 4 objects of Experiment 1. These ANOVAs had two between-subject factors of Condition of familiarization (no familiarization vs. familiarization with 2 objects; no familiarization vs. familiarization to 4 objects) and Order of test presentation (visual array with 2 objects presented first vs. visual array with 4 objects presented first), and two within-subject factors of Test numerosity (2 objects vs. 4 objects) and Test pair (first, second or third).

### Results

**Fig 3:**
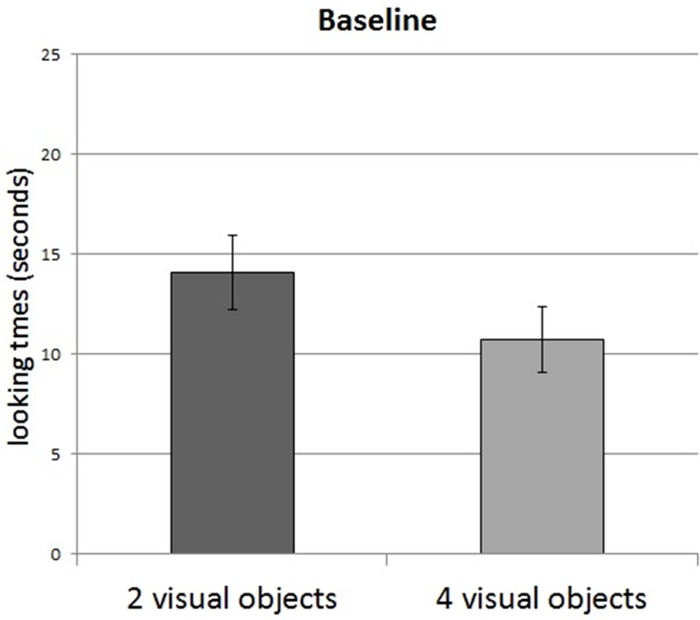
Results of the Baseline Experiment. Infants looked longer at 2 visual objects than at 4 visual objects in the absence of tactile familiarization. Error bars are s.e.m.

The analysis of the Baseline experiment revealed an effect of Test numerosity (F(1,14) = 4.78, p = .04): infants looked longer at the visual array containing 2 objects (m = 14.08 s) than at the array containing 4 objects (m = 10.71 s) (Fig. 3): 10 infants looked longer at the 2-object array, 5 looked longer at the 4-object array, and 1 infant looked equally to both displays (less than 1 s difference). No other significant effects or interaction were observed.

The ANOVA comparing the Baseline to the Familiarization with 2 objects revealed no main effect involving the factor Test numerosity. In particular, there was no interaction between Test numerosity and Condition of Familiarization (p>.1). We nevertheless observed other main effects and interactions. First, an effect of Order of presentation (F(1,28) = 4.75, p = .03) was observed: infants looked generally longer when the visual array containing 2 objects was presented first. Moreover, an effect of Test pair (F(2,56) = 5.67, p = .005), together with an interaction between Test pair and Condition of familiarization (F(2,56) = 4.21, p = .01) showed that infants looked longer at the first test pair when familiarized with 2 objects.

The ANOVA comparing the Baseline to the Familiarization with 4 objects revealed a main effect of Test numerosity (F(1,28) = 18.63, p = .0001): infants looked longer at the visual arrays containing 2 objects, independently of whether they were familiarized with 4 tactile objects or were not familiarized at all. No other main effects or interaction were observed (ps>.09).

### Discussion

The baseline experiment revealed a spontaneous preference for 2 objects over 4 objects, similar to the preference observed after familiarization with 4 objects. Accordingly, there was no interaction on infants’ preference when the baseline group was compared to the group that had been familiarized to 4 objects. However, we did not observe either an interaction between infants’ spontaneous preference at Baseline and infants’ looking after being familiarized with 2 objects. In fact, infants’ behavior in the baseline condition appeared to be halfway between that of the two other groups: 8/16 infants looked longer at the 2-item array than at the 4-item array after touching 2 objects, while 10/16 infants did so in the baseline, and 13/16 infants did so after touching 4 objects. It thus remains possible that both familiarization conditions had an impact on infants’ looking behavior.

In the course of this project, we also tested infants with another pair of small vs. large numerosities, 3 vs. 6. The results confirm the findings of the main experiment. Sixteen infants were tested in each of 2 conditions (14 females, age 4 months and 15 days to 5 months and 0 days; 1 additional infant was excluded because his average looking time exceeded 40 s.): familiarization with three objects, or no familiarization (baseline). Contrary to the main experiment, we did not test a group familiarized with 6 objects, because we feared it would be impossible to design 6 different objects that infants could discriminate tactilely. Again, the total familiarization time was set to 90 s, thus each object (sphere, cube, ring) was presented for 30 s. Visual items were similar to the items presented in the main experiment, again controlling for cumulated area (3-item array: cumulated area = 71.7 cm^2^, individual item area = 23.92 cm^2^; 6-item array: cumulated area = 72.6 cm^2^, individual area = 12.1 cm^2^). As in the main experiment, in the absence of familiarization, infants showed a baseline preference for 3 over 6 objects (F(1,14) = 5.3, p = .03; 11 infants looked longer at the 3-item array, 5 infants looked longer at the 6-item array). This preference vanished after familiarization to 3 tactile objects (F(1,14) = 0.009, p = .92; 9 infants looked longer to 3 items, 5 infants looked longer at the 6-item array and 2 infants showed no preference (less than 1 second difference)), although here again the interaction between Condition of familiarization and Test numerosity failed to reach significance (F(1,28) = 1.61, p = .21). A significant interaction between Baseline vs. Small Number Familiarization (2 or 3), and Test Numerosity (Small vs. Large) was observed when the two experiments were grouped together (F(1,60) = 4.34, p = .04).

Infants’ preference for a small number in the baseline, as well as after familiarization with a large number in the main experiment, indicate that they were able to discriminate between the small and the large visual arrays. However, in itself this preference does not necessarily reflect discrimination of numerosity. For example, the observed preference may be due to some non-numerical differences across arrays, such as individual item size, or summed contour length (cumulative area was controlled across displays). In static displays of dot arrays, infants react only to 4-fold changes in individual area, and not to 3-fold changes as we used here. Nevertheless, this threshold, which was measured over static dot arrays, may not hold for our stimulus. Alternatively, since the format of our stimuli emphasized individual objects (real objects displaying independent oscillating movements), it is also possible that both our small and large arrays engaged automatically object tracking representations. In line with this hypothesis, we often observed that infants extended their arms and attempted to grasp the displays. Under this second interpretation, two- or three-item stimuli would be preferred because they fit with object tracking representations, unlike 4- or 6-item stimuli.

Interestingly, in previous research using similar stimuli, infants did not show a spontaneous preference between 2 or 3 objects [7]. This result is compatible with both of the interpretations offered above. On one hand, both arrays were within object tracking range in the 2 vs. 3 experiment, unlike in the present study: the hypothesis based on array size predicts no preference in this case. On the other hand, because stimuli were controlled on summed area in both studies, non-numerical parameters were less discriminable in the 2 vs. 3 experiment than in our study. If the preference in the 2 vs. 4 baseline experiment was due individual item size, summed contour length, or any other quantitative parameter, absence of such preference in the 2 vs. 3 baseline experiment may be due to a failure to discriminate the relevant parameter(s).

## General Discussion

Several studies showed infants failing to compare a small vs. a large numerosity, a dissociation that was taken as the hallmark signature for the existence of two separate systems [4–8–10–11–17]. In some situations, however, infants can succeed in discriminating a small vs. a large numerosity, for example if the ratio between the two numerosities largely surpasses the usual ratio for ANS representations at a given age [10], if the attentional load is high [16], or if the format of stimuli does not trigger parallel tracking mechanisms [18].

In the present study, we tested whether a paradigm involving cross-modal transfer could incite infants to access numerosity representations in a more abstract format, common between the small and large ranges. We used an attested paradigm, where infants were shown to transfer numeric information about small sets of 2 or 3 objects from touch to vision [7]. However, when infants were tested with a contrast between a small and a large numerosity (2 vs. 4), the pattern of results was partially different. Infants generally showed a preference for the visual array containing a new number of objects, as they had done with sets of 2 vs. 3 objects, but in detail this pattern was present only in the group that had been familiarized with 4 objects, not in the group familiarized with 2 objects. In addition, in a baseline experiment we observed that, in the absence of a tactile familiarization, infants spontaneously displayed a preference for 2-object over 4-object arrays.

Did infants succeed at transferring representations of small and large numerosities across the tactile and visual modalities? For infants familiarized with two objects, previous evidence indicates that infants can extract the numerosity from our tactile stimuli and later recognize the same numerosity in 2 visual objects, when contrasted with 3 [7]. Two interpretations can be given to explain infants’ failure to prefer 4 over 2 visual objects in the present research. On one hand, infants may have failed to compare visual stimuli across the small and large ranges of numerosity, thus converging towards the attested dissociation between small and large ranges. On the other hand, it is possible that infants were sensitive to the mismatch between the 2 tactile objects and the 4-item arrays, but the preference for the novel numerosity (4) was counteracted by the spontaneous preference observed at baseline.

The situation is less clear in the second condition of familiarization (with 4 objects). On one hand, we observed a preference for the novel numerosity following familiarization with 4, together with an interaction contrasting infants’ preferences after familiarization with 2 vs. with 4. Generally, the data may thus reveal a general preference for novelty, which, depending on the condition, would either be reinforced or counteracted by infants’ spontaneous preference for 2 over 4 objects. In line with this interpretation, our baseline results appear to be intermediate between both conditions of familiarization. Note however that, ideally, this pattern of results should have yielded interactions between each experimental and baseline conditions, thus this interpretation is only offered as tentative. Nonetheless, our results are compatible with the hypothesis that infants displayed a continuous response across the small and large numerosity ranges.

However, it is also possible that the preference for 2 was not induced by the familiarization phase, but reflected infants’ spontaneous preference for 2 over 4, as demonstrated in the baseline condition. Under this interpretation, infants failed to transfer information about numerosity when familiarized with 4 tactile objects, and this could be due to different reasons. First, infants may have failed to process the numerosity of the tactile familiarization. Because objects were presented sequentially, our stimuli may have encouraged infants to engage objects tracking resources, as is the case for items presented sequentially in the visual modality [9–2]. As a result, just like in the visual modality, infants would have failed to represent any numerical information from 4-object sets. Alternatively, infant may have succeeded to compute the numerosity of 4 tactile objects, but failed to represent this information in the format of analogue magnitudes, or any format compatible with both the tactile and visual modalities. Indeed, while ample evidence shows that infants represent visual or auditory numerosities via ANS representations, we do not know of any evidence showing that the tactile modality can yield ANS representations. This explanation, although theoretical possible, seems rather unlikely given that ANS representations are accessed from a wide range of stimuli, from visual, to auditory, or kinesthetic. Lastly, it is possible that infants represented the numerosity of the 4 tactile objects in the format of the ANS, yet failed to transfer this information when faced with the visual stimuli. Indeed, in all previous research using bimodal (audiovisual) paradigms with large approximate numerosities, the stimuli were presented concomitantly in both modalities [4–22], or the testing phase was preceded by a familiarization phase linking together the audio and visual stimuli [23]. In the absence of such link, infants aged 5-months and beyond may not be inclined to compare ANS representations across senses.

In conclusion, in general our results showed an impact of touching 2 vs. 4 objects on infants’ subsequent visual preference between 2- and 4-item visual arrays. These results are compatible both with the hypothesis that infants engaged in dissociated representations for the small and large numerosity ranges, and with the hypothesis that 5-month-old infants were able to discriminate a small vs. a large numerosity. It is thus unclear whether using a bimodal paradigm helped infants to extract the numerical information in a range-independent format. Further research is needed to disentangle these possibilities.
